# Synchrotron Phase Tomography: An Emerging Imaging Method for Microvessel Detection in Engineered Bone of Craniofacial Districts

**DOI:** 10.3389/fphys.2017.00769

**Published:** 2017-09-29

**Authors:** Alessandra Giuliani, Serena Mazzoni, Luigi Mele, Davide Liccardo, Giuliana Tromba, Max Langer

**Affiliations:** ^1^Sezione di Biochimica, Biologia e Fisica Applicata, Dipartimento di Scienze Cliniche Specialistiche e Odontostomatologiche, Università Politecnica delle Marche, Ancona, Italy; ^2^Sezione di Biotecnologie, Istologia Medica e Biologia Molecolare, Dipartimento di Medicina Sperimentale, Università degli Studi della Campania “L. Vanvitelli”, Naples, Italy; ^3^Elettra Sincrotrone Trieste S.C.p.A, Trieste, Italy; ^4^Centre de Recherche en Acquisition et Traitment d’Images pour la Santé (CREATIS), Centre National de la Recherche Scientifique (CNRS) UMR 5220, Institut national de la santé et de la recherche médicale (Inserm) U1206, Université de Lyon, INSA-Lyon, Villeurbanne, France

**Keywords:** phase tomography, synchrotron radiation, microvessels, craniofacial bone engineering, X-ray phase-contrast imaging

## Abstract

The engineering of large 3D constructs, such as certain craniofacial bone districts, is nowadays a critical challenge. Indeed, the amount of oxygen needed for cell survival is able to reach a maximum diffusion distance of ~150–200 μm from the original vascularization vector, often hampering the long-term survival of the regenerated tissues. Thus, the rapid growth of new blood vessels, delivering oxygen and nutrients also to the inner cells of the bone grafts, is mandatory for their long-term function in clinical practice. Unfortunately, significant progress in this direction is currently hindered by a lack of methods with which to visualize these processes in 3D and reliably quantify them. In this regard, a challenging method for simultaneous 3D imaging and analysis of microvascularization and bone microstructure has emerged in recent years: it is based on the use of synchrotron phase tomography. This technique is able to simultaneously identify multiple tissue features in a craniofacial bone site (e.g., the microvascular and the calcified tissue structure). Moreover, it overcomes the intrinsic limitations of both histology, achieving only a 2D characterization, and conventional tomographic approaches, poorly resolving the vascularization net in the case of an incomplete filling of the newly formed microvessels by contrast agents. Indeed, phase tomography, being based on phase differences among the scattered X-ray waves, is capable of discriminating tissues with similar absorption coefficients (like vessels and woven bone) in defined experimental conditions. The approach reviewed here is based on the most recent experiences applied to bone regeneration in the craniofacial region.

## Introduction

Nowadays, craniofacial bone defects due to congenital conditions, disease, and injury cause major clinical issues, often solved with tissue replacement by autologous grafting. However, sometimes this procedure is hampered by an extensive donor site morbidity. In these cases, bone engineering protocols could support restoration of the function, or replace damaged or diseased tissues (Alsberg et al., 2001).

Since the long-term function of three-dimensional (3D) bone substitute biomaterials (BSB) is strongly dependent on adequate vascularization after grafting, research in craniofacial bone engineering has recently focused on approaches involving angiogenesis (Auger et al., 2013). These include the delivery of different growth factors to the defect site (Yoo and Kwon, 2013) or the engineering of microvascular networks by using endothelial cells and stem cells (Liu et al., 2013). Nevertheless, current vascularization methods are often not sufficiently rapid for an adequate cellular oxygen supply. Therefore, it is necessary to create microvascular networks within 3D tissue constructs *in vitro* before grafting (Laschke et al., 2006).

However, full comprehension of the bone vascularization pathways is still hampered by limitations in the use of imaging techniques to monitor these processes *in-vivo*. Nowadays, new and interesting imaging modes are available. Upputuri et al. (2015) recently classified the vascular imaging approaches into three groups: non-optical techniques (X-ray, magnetic resonance, ultrasound, and positron emission imaging), optical techniques (optical coherence, fluorescence, multiphoton, and laser speckle imaging), and hybrid techniques (photoacoustic imaging). Physical origin of the contrast, levels of resolution achieved, imaging depth and structures resolved (microvessels, bone, etc.) have been described and summarized for each group.

Inside the non-optical group, great interest is focused on X-ray imaging methods. They are based on X-ray attenuation of the different tissues and have been successfully used over the past few years to visualize large blood vessels.

In this regard, high resolution tomography (microCT) (Arkudas et al., 2010; Barbetta et al., 2012) is able to provide much higher resolution (~1 μm) imaging, than ultrasound (~30 μm) and MRI (~100 μm), allowing the visualization and quantification of microvasculature. This procedure is normally achieved with the use of contrast agents, which are radio opaque and radio dense fillers. It was recently proved by this approach that the 10 μm medium resolution microCT is able to image with success medium and large blood vessels (Nebuloni et al., 2014). Moreover, Langer et al. (Langer et al., 2011) successfully imaged the microvasculature with contrast agent in rat and mouse bone. While they proved that parts of the connectivity were not preserved (due to partial volume effect), a very large part of the contrast agent volume was retrieved.

Also, in recent years, a new method for combined 3D imaging and analysis of microvascularization and bone microstructure has emerged. It is based on the use of synchrotron phase microtomography (PhC-microCT) and it allows, as opposed to conventional 2D techniques like histology, to simultaneously identify in 3D multiple tissue features without using contrast agents. This is due to the increased sensitivity of phase sensitive X-ray imaging techniques. Indeed, due to increased sensitivity, this technique also overcomes the intrinsic limitations of conventional tomographic approaches, often unable to reliably reconstruct the full vascularization network in case of incomplete filling of microvessels by contrast agents (Fei et al., 2010).

In this mini-review, we briefly discuss the very recent advances in synchrotron phase tomography aiming at high-resolution imaging of engineered bone vasculature in craniofacial districts.

## Approaching synchrotron phase tomography methods

Differently from the conventional (attenuation-based) microCT in which the contrast is due to attenuation differences within the sample, in PhC-microCT the contrast originates from the phase shift of the X-ray beam passing through the matter (Snigirev et al., 1995; Bravin et al., 2013). This phase shift in non-mineralized biological tissues can be up to three orders of magnitude larger than attenuation (Momose et al., 1996; Lewis et al., 2003), explaining the highly increased contrast that has been observed with PhC-microCT imaging in investigating esophagus (Lewis et al., 2003), brain (Connor et al., 2009; Pinzer et al., 2012; Marinescu et al., 2013), liver (Lewis et al., 2003; Herzen et al., 2014), kidney (Velroyen et al., 2014), lung (Lewis et al., 2003), cartilage (Coan et al., 2010; Marenzana et al., 2012; Horng et al., 2014) and breast tissues (Arfelli et al., 2000; Stampanoni et al., 2011).

At least three methods have been studied for phase contrast X-ray imaging: X-ray grating interferometry (Momose, 1995, 2003; Momose et al., 1996), diffraction enhanced imaging (Davis et al., 1995; Ingal and Beliaevskaya, 1995; Chapman et al., 1997, 1998) and propagation-based imaging (Snigirev et al., 1995; Wilkins et al., 1996; Cloetens et al., 1999).

The first two methods are often confined to synchrotron sources because they require a monochromatic and highly collimated x-ray beam. Indeed, when coherence grating is used at conventional x-ray sources, a detrimental flux reduction is experienced. In turn, this flux reduction would require longer exposure times than at synchrotrons, hampering, as a consequence, the translation of the method to clinical settings (Nesch et al., 2009).

Very recently, grating-based (GB) Talbot interferometry (Weitkamp et al., 2005; Momose et al., 2009) was successfully applied, combined to conventional polychromatic x-ray sources. A third grating (Pfeiffer et al., 2007) was used to study weakly absorbing samples and, using synchrotron facilities, to excellently discriminate soft-tissues in cancerous human liver tissue (Noel et al., 2013) and in atherosclerotic plaques (Hetterich et al., 2013). Other studies performed with polychromatic x-ray sources allowed to achieve an improved imaging of parenchymal lung disease (Yaroshenko et al., 2013), breast lesions (Hauser et al., 2013; Sztrokay et al., 2013), cartilage (Tanaka et al., 2013), atherosclerotic plaques (Saam et al., 2013), and renal ischemia (Velroyen et al., 2014).

Unlike X-ray interferometry, the diffraction enhanced imaging method exploits the benefits of a μrad angular resolution achieved by filtering X-rays deflected by refraction. This is made possible using an analyzer crystal after the sample to select refracted X-rays from the sample.

Instead, the propagation-based method generates contrast by Fresnel or Fraunhofer diffraction: the first is achieved by placing the detector and the sample at a moderate distance.

In practice, at lower resolutions, the X-ray interferometric imaging is thus the preferential approach for revealing soft structures, being able to also sense the shallower phase gradients, compared to the diffraction enhanced and propagation-based imaging methods. This is due to the principles of contrast generation; stronger contrast is generated at structural boundaries where the variations of refractive index are larger due to rapid phase gradients (Momose, 2003).

On the other hand, the propagation-based imaging method is of great advantage in high-resolution imaging because no optical components are needed when a coherent X-ray source is available, like at synchrotron facilities. The chance to get high resolutions has made propagation-based imaging the technique of choice in the study of both human bone ultrastructure and vasculature, with focused pre-clinical and clinical studies on vessels of medium and small thickness.

Concerning the investigation of human bone ultrastructure, X-ray phase nano-tomography, based on the propagation method, was recently shown to provide the appropriate spatial resolution and sensitivity to efficiently visualize and quantify the 3D organization of the lacuno-canalicular pattern (Langer et al., 2012). The study was performed considering several cells in osteonal and interstitial tissue: nanoscale density variations revealed that the cement line separating these tissues is hypermineralized. Moreover, the organization of the collagen fibers was reconstructed in 3D, showing a twisted plywood structure.

Imaging brain microvasculature is crucial for plasticity studies of cerebrovascular diseases. Traditionally, in the brain, absorption-based microCT and microMRI methods are applied, using contrast agents for a better visualization of the vasculature.

Very recently, propagation-based synchrotron PhC-microCT was applied to visualize the whole mouse brain microvasculature. It was carried out at high resolution (~3.7 μm) and without contrast agents (Miao et al., 2016). Microvasculature changes in C57BL/6 mice brain (*n* = 14) after 14-day reperfusion from transient middle cerebral artery occlusion (tMCAO) were observed. PhC-microCT demonstrated that the branching radius ratio (post- to pre-injury) of small vessels (radius < 7.4 μm) in the injury group was significantly smaller than in the sham group. This result revealed active angiogenesis in brain recovery after stroke.

In another recent study, Fratini et al. (2015) showed that PhC-microCT allows the simultaneous visualization of three-dimensional micro-vascular network and neuronal systems of *ex-vivo* mouse spinal cord. This experiment was carried out at scales spanning from millimeters to hundreds of nanometers, without contrast agents and destructive sample preparation. Images of both the 3D distribution of micro-capillary network and the micrometric nerve fibers, axon-bundles and neuron soma, were obtained, confirming the efficiency of this technique also in pre-clinical studies of neurodegenerative pathologies and spinal-cord-injuries.

## Assessment of bone microvessels detection by phase tomography

Conventional X-ray microCT is a technique that allows a good visualization of the structure of mineralized bone and biomaterials, but it fails when attempting to discern soft tissues at high resolutions. On the contrary, PhC-microCT, based on propagation-based settings, was demonstrated to present, at high resolution, a better soft tissue contrast than conventional CT, clearly discriminating ligamentous, muscular, neural, and vascular structures (Horng et al., 2014).

These findings led some authors to investigate the 3D vascularization of engineered-bone tissue. Detailed imaging and a quantitative description of the complete vascular network in such constructs is crucial for monitoring the relation between bone formation and vascularization and phase tomography was shown to efficiently discriminate tissues with similar absorption coefficients (Langer et al., 2009).

However, the previous study was still limited by the use of a contrast agent. Indeed, although a new phase-contrast medium—the micro-bubble—was recently successfully applied to angiography applications (Tang et al., 2011), different authors agree that PhC-microCT is able to perform a 3D visualization of the smallest capillaries (Momose et al., 2000; Fratini et al., 2015) in mice, without the use of any contrast agent.

This fact was confirmed in another recent study (Bukreeva et al., 2015), where synchrotron PhC-microCT was applied to visualize and analyze the 3D micro-vascular networks in bone-engineered constructs, made of porous ceramic scaffolds loaded with bone marrow stromal cells (BMSC), in an ectopic bone formation mouse-model. Samples seeded and not seeded with BMSC were compared, with or without the use of contrast agents. The authors achieved the 3D distribution of both vessels and collagen matrix and obtained quantitative information for the different samples, even for those not stained.

### Experience in craniofacial districts

Nowadays, autologous bone is still considered the ideal grafting material in the craniofacial district (Yamanichi et al., 2008; Iezzi et al., 2017). Autologous grafts are vascularized and contain viable osteoblasts, organic and inorganic matrices, and growth factors that allow remodeling and structural integration with the host site. However, there are significant limitations associated with the use of autologous grafts, including the availability of donor tissue (since it has to be obtained intraorally), the need of additional surgical procedures and, consequently, increased operating times and costs (Tsigkou et al., 2010; Iezzi et al., 2017). When directly using synthetic and allogeneic grafts, only the periphery of the graft is efficiently vascularized. As a consequence, a central zone of necrosis frequently occurs, resulting in a shell of ossification at the surface, but with low level penetration, due to the limited transport of oxygen and metabolic requirements to the inner cell mass (Tsigkou et al., 2010). Thus, a rapid vascularization of bone grafts is a clinical challenge, based on the fact that the development of a mature and functional vasculature not only depends on migration and proliferation of endothelial cells but also requires cooperation and symbiosis between them and perivascular cells, acting as a potent stabilizer of the engineered blood vessels formed in the porous bone scaffold (Tsigkou et al., 2010).

Therefore a major task is to find advanced imaging techniques that could verify and quantify the mineralization process and neovascularization of grafts, at the early stages of bone formation (Paino et al., 2017). Third-generation synchrotron facilities produce brilliant X-ray photon beams, having high spatial coherence properties. They have been demonstrated to be suitable for several tissue engineering studies, detecting *in-vitro* the newly formed extracellular matrix (Albertini et al., 2009), the early colonies of endothelial cells (Giuliani et al., 2014), and the early phases of bone mineralization (Manescu et al., 2016; Mazzoni et al., 2017), by introducing X-ray imaging methods based on phase-contrast.

**Figure 1:**
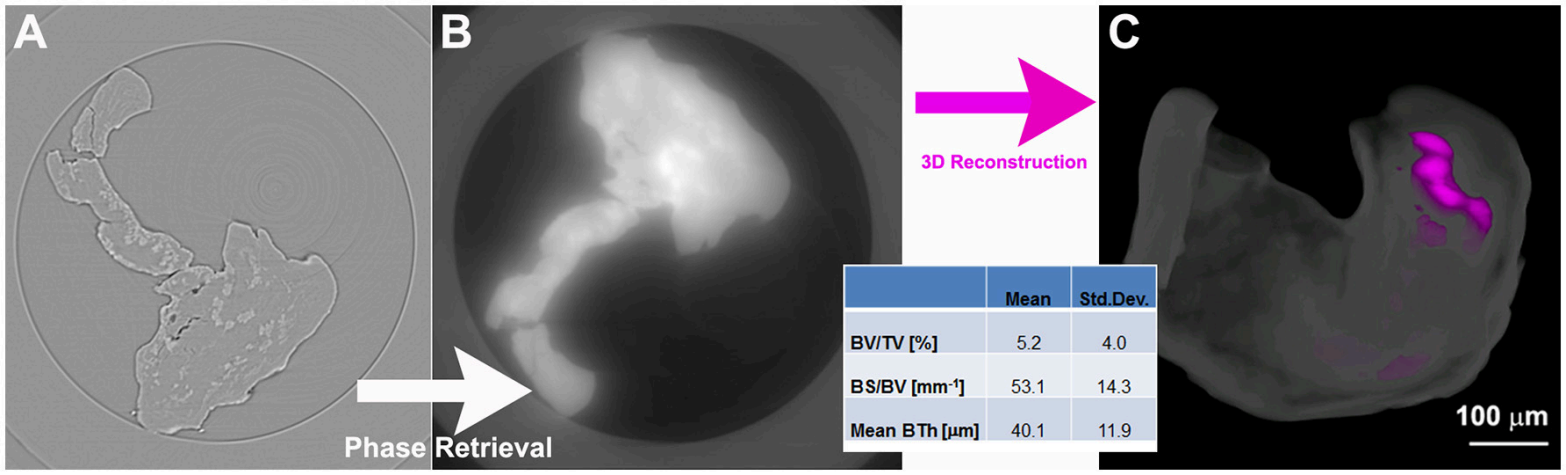
Phase-contrast microCT analysis of woven bone (WB). **(A)** 2D slice before phase-retrieval processing: the edge-enhancement signal prevents a reliable discrimination and quantification of the two phases (WB and newly mineralized bone). **(B)** The same 2D slice as in **(A)** after phase-retrieval processing; and **(C)** 3D reconstruction of a WB sample after processing by phase retrieval: the woven structure is shown in translucent white, whereas the newly formed mineralized bone is depicted in magenta; (bottom inset) morphometric analysis of the mineralized bone. This figure was originally published in Paino et al. (2017; www.clinsci.org/content/early/2017/02/16/CS20170047).

Very recently, some authors (Paino et al., 2017) showed that human dental pulp stem cells (hDPSCs) can lead to a bone tissue ready to be grafted for clinical application. They demonstrated that hDPSCs proliferate *in-vitro*, differentiate into osteoblasts and express genes associated with angiogenesis factors, such as VEGF and PDGFA. Synchrotron-based X-ray phase-contrast imaging proved that, after 40 days of culture in standard medium, hDPSCs formed woven bone (WB), i.e., fibrous bone with a low level of mineralization. In particular, the PhC-microCT analysis was performed using a polychromatic beam, with a sample-to-detector distance of 150 mm, corresponding to a single-distance phase-contrast set-up. The osteogenic potential of WB fabricated after *in vitro* hDPSC culture was validated by the quantitative data extracted from the 3D PhC-microCT analysis (Figure 1). However, it was not possible to detect any vascularization using this technique.

**Figure 2:**
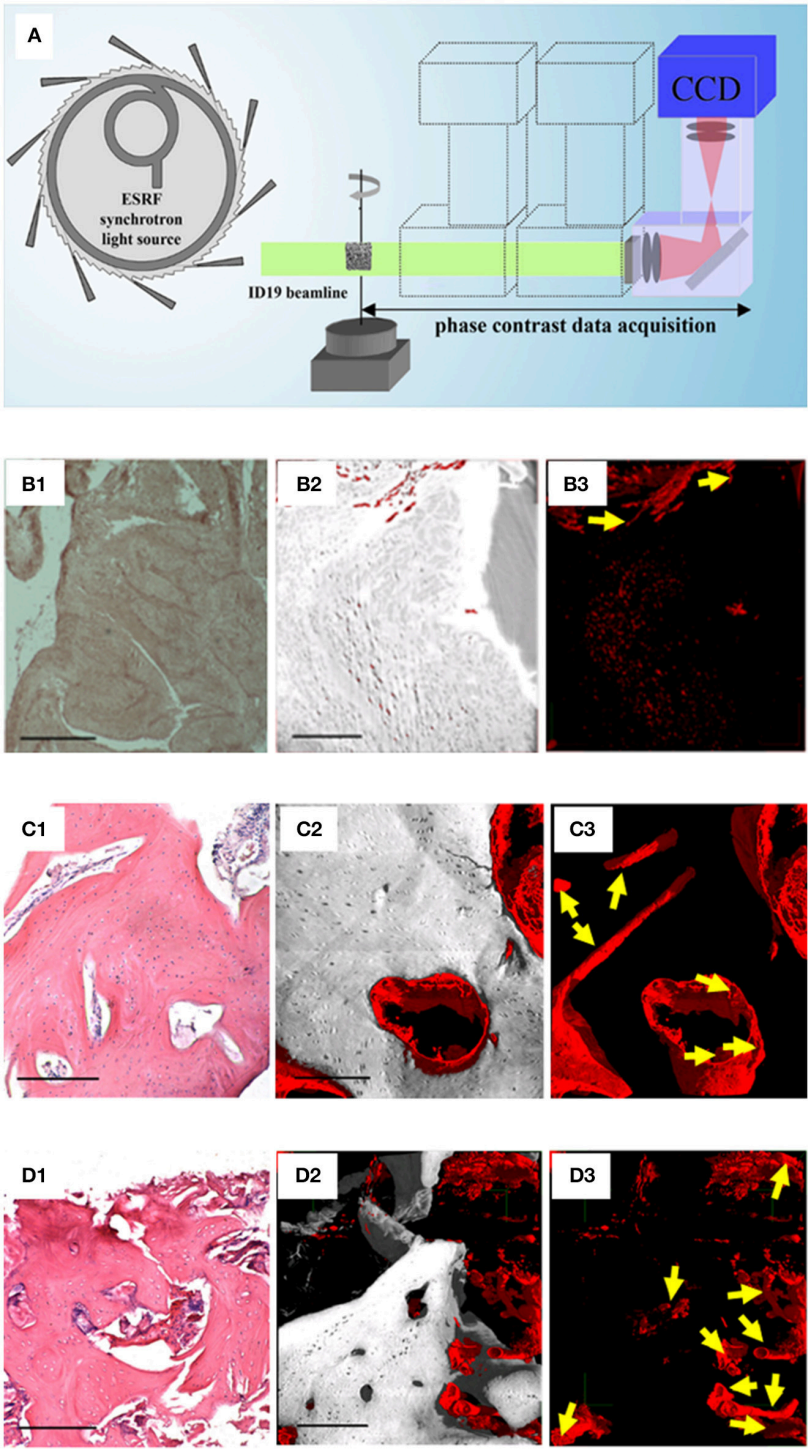
Phase-contrast Holotomography (HT). **(A)** HT set-up at the ID19 beamline of the European Synchrotron Radiation Facility. Histological and HT analysis of the WB **(B)**, of a human *in vivo* stem cell-treated mandible **(C)**, and of a human mandible control **(D)**. For each group **(B–D)**, the panels on the left (1) represent histological sections with H&E staining, as reference; the central (2), and the right (3) panels are subvolumes of the 3D HT reconstructions where, to improve visualization, all phases were virtually deleted except for bone and vessels (2), or exclusively except for vessels (3). Yellow arrows indicate portions of vessels to discriminate them from possible artifacts. Scale bars = 250 μm. Panel **(A)** was originally published in Giuliani (2016; https://doi.org/10.1016/B978-0-08-100287-2.00012-4); Panels of the group-B were originally published in Paino et al. (2017; www.clinsci.org/content/early/2017/02/16/CS20170047); Panels of the group-C and of the group-D were originally published in Giuliani et al. (2013; http://onlinelibrary.wiley.com/doi/10.5966/sctm.2012-0136/epdf).

In this regard, the holotomography (HT) technique added fundamental information (Paino et al., 2017). The HT approach differs from the PhC-microCT method based on a single distance in that the acquisition consists of tomographic scans at four different propagation distances, followed by a different reconstruction algorithm (Figure 2A). The HT analysis allowed to achieve a 3D reconstruction of the WB (Figures 2B1,B2), assessing the presence of new vessels (Figure 2B3). Indeed, because of their low attenuation coefficient, these new vessels are transparent in conventional attenuation-based tomographic reconstructions and the PhC-microCT method based on a single distance was still found to be not sufficiently sensitive. Indeed, this HT evidence confirmed deductions derived from the fact that hDPSCs strongly expressed high levels of VEGF and PDGF-A, which explains the vessel formation in the WB. This last finding is of paramount interest for physiology of the bone in craniofacial districts, because it satisfies the need of neoangiogenesis in the engineered site. Thus, these results strongly support the rationale that hDPSCs possess significant differentiation capabilities toward osteoangiogenesis, matching the gold standard for obtaining well-vascularized bone (Paino et al., 2017).

Moreover, some studies (d'Aquino et al., 2009) demonstrated that hDPSCs differentiate into osteoblasts and, when seeded on collagen I scaffolds, efficiently contribute to repair human mandible defects. In this context, another study (Giuliani et al., 2013) showed, by synchrotron HT, the stability and quality of both the regenerated bone and the vessel network 3 years after grafting. It was found that the regenerated tissue in grafted sites was unexpectedly constituted by compact bone, structurally different and denser compared to the healthy native alveolar bone of the same patient. However, in both the human mandible control and the human *in vivo* SC-treated mandible biopsies analyzed, in agreement with histological analyses, after 3 years the regenerated bone was well structured and vascularized. Two subvolumes of the human *in vivo* SC-treated mandible are shown in Figures 2C1,C2: one, referring to a representative histological slice, was used as a reference (Figure 2C1); the other, referring to the 3D reconstruction of the HT scan and the subsequent data analysis (Figures 2C2,C3). The unmineralized phases, with the exception of the one representing vessels, were virtually suppressed for a better visualization of bone (gray) and its vascularization (red). As confirmed histologically (Figure 2C1) these 3D reconstructions demonstrate that after 3 years bone tissue was well structured and vascularized. In particular, in Figure 2C3, only densities compatible with the vessel phase have been visualized, clearly showing a good vascularization. The same information is represented in Figures 2D1–D3 for human mandible control. It is clearly shown that the bone is a well-structured cancellous structure (Figures 2D1,D2), with an homogeneous and fully organized vessel network (Figure 2D3): although here the vascularization is more structured than in the human *in vivo* SC-treated mandible, density signals compatible with neo-vessels were found in several areas of the biopsy retrieved from the treated site, confirming *in-vivo* that the hDPSCs have significant differentiation capabilities toward osteoangiogenesis (Giuliani et al., 2013).

## Conclusions

While PhC-microCT was demonstrated to be capable of visualizing the 3D vessel network in *in-vitro* and *ex-vivo* conditions and without any sample sectioning and preparation, the use of coherent and highly brilliant Synchrotron X-ray sources was mandatory in order to achieve a higher image quality with sub-micrometer spatial resolution. Indeed, as reported in the literature and summarized in Table 1, microvessel detection in engineered bone was carried out mainly in two ways: by attenuation-based microCT, with the use of contrast agents, or by propagation-based PhC-microCT, without any marker. However, the application of the last method has required, up to now, access to synchrotron facilities. This fact constitutes a relevant limitation for a possible future use of phase-contrast imaging in clinical practice, since the radiation dose would be too high. Therefore, we propose this approach as a fundamental tool for angiogenesis studies in preclinical research and bone post-extractive studies in craniofacial districts.

**Table 1:** Summary of high resolution tomography (microCT) imaging modalities for vascular imaging in tissue engineering. Image resolution up to 600 nm (in propagation-based settings).

	**Modality**	**Anatomical structures resolved**	**Advantages/disadvantages**
Attenuation based	Without contrast agents	Mineralized tissues (bone, enamel, dentin, etc.)	Good 3D imaging of the mineralized structures/non-mineralized tissues not discriminated^*^
	With contrast agents	Blood vessels, cells/stem cells	Successful imaging of medium and large blood vessels/part of connectivity lost (no detection of small vessels)^**^
Phase-contrast based	Grating interferometry	Cancerous human liver tissue, atherosclerotic plaques, parenchymal lung, breast lesions, cartilage and renal ischemia^§^	Contrast-agent-free method, excellent for weakly absorbing samples imaging^§^/detrimental flux reduction and longer exposure times^§§^
	Diffraction enhanced	Breast tissues, cartilage, brain^#^	Contrast-agent-free method, μrad angular resolution/confined to synchrotron sources, not effective in sensing shallow phase gradients^##^
	Propagation-based (single distance)	First phases of bone mineralization, blood vessels, ligamentous and muscular structures nerve fibers, axon-bundles and neuron soma^+^	Contrast-agent-free method, high-resolution imaging/confined to synchrotron sources, not effective in sensing shallow phase gradients^##^
	Propagation-based (multiple distance)	Bone ultrastructure, small blood vessels^++^	Contrast-agent-free method, very high-resolution imaging, successful imaging of small blood vessels/confined to synchrotron sources^##^

* *Arkudas et al., 2010; Barbetta et al., 2012*.

** *Fei et al., 2010; Langer et al., 2011; Nebuloni et al., 2014*.

§ *Hauser et al., 2013; Hetterich et al., 2013; Noel et al., 2013; Saam et al., 2013; Sztrokay et al., 2013; Tanaka et al., 2013; Yaroshenko et al., 2013; Velroyen et al., 2014*.

§§ *Nesch et al., 2009*.

# *Chapman et al., 1998; Connor et al., 2009; Coan et al., 2010*.

## *Momose, 2003*.

+ *Horng et al., 2014; Bukreeva et al., 2015; Fratini et al., 2015; Manescu et al., 2016; Miao et al., 2016; Mazzoni et al., 2017*.

++ *Langer et al., 2012; Giuliani et al., 2013; Paino et al., 2017*.

## Author contributions

AG: Concept and design; revision of the whole literature; coordination of the work drafting; final version definition and approval. SM and GT: Design (phase-contrast tomography); data research in literature on PhC-microCT; work drafting; final version approval. LM and DL: Design (physiology concepts); data research in literature on physiological interpretation of data; work drafting; final version approval. ML: Concept and design; data research in literature on PhC-microCT; work drafting; final version definition and approval. All the authors agreed to be accountable for all aspects of the work in ensuring that questions related to the accuracy or integrity of any part of the work are appropriately investigated and resolved.

### Conflict of interest statement

The authors declare that the research was conducted in the absence of any commercial or financial relationships that could be construed as a potential conflict of interest. The reviewer CA and handling Editor declared their shared affiliation.
